# Long-term outcome of a randomized controlled universal prevention trial through a positive parenting program: is it worth the effort?

**DOI:** 10.1186/1753-2000-4-14

**Published:** 2010-05-16

**Authors:** Kurt Hahlweg, Nina Heinrichs, Annett Kuschel, Heike Bertram, Sebastian Naumann

**Affiliations:** 1 Technical University Braunschweig, Department of Clinical Psychology, Psychotherapy, and Assessment, Humboldtstr. 33, 38106, Germany; 2 University of Bielefeld, Faculty for Psychology and Sports Science, Department of Clinical Child and Adolescent Psychology and Psychotherapy, Postfach 10 01 31, D-33501 Bielefeld, Germany

## Abstract

**Background:**

Approximately 20% of children experience internalizing or externalizing DSM-IV-TR disorders. This prevalence rate cannot be reduced through treatment only. Effective preventive interventions are therefore urgently needed. The aim of the current investigation is to evaluate the two-year efficacy of the group Triple P parenting program administered universally for the prevention of child behavior problems.

**Methods:**

Based on their respective preschool, *N *= 280 families were randomly assigned either to the parent training or to the control group. The efficacy was analyzed using multi-source assessments, including questionnaires by mother and father, behavioral observation of mother-child interaction, and teacher evaluations.

**Results:**

At the 2-year follow-up, both parents in the Triple P intervention reported significant reductions in dysfunctional parenting behavior, and mothers also an increase in positive parenting behavior. In addition, mothers reported significant reductions in internalizing and externalizing child behavior. Single-parent mothers in the Triple P intervention did not report significant changes in parenting or child problem behavior which is primarily due to inexplicable high positive effects in single parent mothers of the control group. Neither mother-child interactions nor teacher ratings yielded significant results.

**Conclusions:**

The results support the long-term efficacy of the Triple P - group program as a universal prevention intervention for changing parenting behavior in two-parent households, but not necessarily in single-parent mothers.

## Background

Behavioral and emotional disturbances are very common among children and adolescents. Approximately 20% of children in western, industrialized countries experience the signs and symptoms that constitute internalizing (e.g. anxiety/depression, withdrawal) or externalizing (e.g. oppositional defiance, aggression) DSM-IV disorders [1]. Left untreated, externalizing disorders in childhood tend to persist and evolve into more antisocial behaviors in adulthood [2]. Similarly, childhood internalizing disorders place these individuals at higher risk for persistent anxiety and depressive disorders in adolescence and adulthood [3]. In addition to the costs of treating such problems, social costs include school dropout, unemployment, family breakdown, drug and alcohol misuse, and increased delinquency and risky behaviors [4].

Examining the effects of prevention programs on the incidence of mental disorders is one of the most important research questions for mental health prevention. Mental disorders account for 22% of the total burden of disease, as measured in disability-adjusted life years lost [5]. Effective prevention programs may potentially contribute to the reduction of this enormous burden of mental disorders. It is estimated that only half of the burden of the common mental disorders can be averted with existing treatment methods (both psychological and pharmacological) given maximized coverage (the number of people seeking treatment), clinician competence, and patient compliance to treatment [6,7]. Whereas there exists a variety of evidence-based treatments for many child behavior problems (e.g., drug treatment, psychotherapy, and parenting programs; [8,9]), only few children who need these treatments can access them [1]. In Canada, only one out of five children with a psychological disorder has any contact with mental health service [10]. Evidence-based treatments are generally costly, time consuming, and require intensively trained professionals to be delivered. "Given that treatment services can never hope to meet the needs of all children with mental health problems, prevention is an essential first step in a public health approach" [[4], p. 318].

The life-course persistent pathway from childhood to adult disorders may be best interrupted early in life, when these behavioral patterns are more easily modified [11]. Family risk factors, such as a lack of a positive relationship with parents, insecure attachment, harsh or inconsistent discipline practices, marital problems, and parental psychopathology increase the risk that children will develop major behavioral and emotional problems [12,13].

The important mediating role of parenting for child behavior problems is well-established and has led to the development of a variety of parenting interventions. Parent Training (PT), derived from social learning, functional analysis, and cognitive-behavioral principles, is considered the intervention of choice for treatment and prevention of conduct problems in young children [14,15]. Parents typically are taught to increase positive management skills such as providing praise, positive attention, or physical affection and to reduce coercive and inconsistent parenting practices by using consistent and firm discipline. Positive effects have been replicated many times across different studies, investigators, and countries, and with a diverse range of client populations [16-19]. In the latest meta-analysis of 77 primary efficacy studies of PT-programs by Kaminski et al. [14], an overall inter-group mean effects size (Cohens d) of 0.34 was found (CI = 0.29 - 0.39; range = -0.61 - 3.69). Specifically, the mean effect sizes for parenting measures were 0.43, for child externalizing behaviours 0.25, for child internalizing behaviours 0.40, and for child social competence 0.13, respectively.

The Triple P-Positive Parenting Program developed by Sanders and colleagues [18] is an example of a population-based, multilevel approach to parenting intervention, based on the above mentioned principles. The Triple P system has five different levels of support for parents in raising children, and it involves a number of different delivery modalities including individual, group, telephone-assisted, and self-directed programs. This public health perspective involves identifying the minimally sufficient conditions that need to change to alter at-risk children's developmental trajectories for developing serious conduct problems and make these interventions broadly available to parents. The Triple P system is widely spread internationally and has been well evaluated. A recent meta-analysis by Nowak and Heinrichs [20] included 55 Triple P intervention studies reporting outcome data. The mean inter-group effect size (Cohen's d) across intervention levels was 0.38; specifically 0.38 and 0.35 for parenting and child behavior problems, respectively. One of the few limitations of these studies is the lack of long-term controlled outcome investigations. This may be primarily due to the frequently employed wait-list control design in previously published studies.

Whereas the efficacy of PT for children at risk because of their exposure to social or familial risk factors (selective prevention) and for subclinical (indicated prevention) or DSM-IV-TR diagnosed children seems to be established, at present only five randomized controlled trials using a *universal *prevention approach (intervention is offered to all parents) with *preschool *children have been published. Eisner, Ribeaud, Juenger, and Meidert [21] recruited over 1.000 families in Zurich, Switzerland, and randomized them either to the Triple P parent-training or to a control group. About 14-18 month later, Triple P families showed a significant reduction in corporal punishment and impulsive parenting, and a stabilizing effect on the family climate while families in the control group deteriorated. Other parenting behaviours did not significantly change, however. Also, based on teacher ratings, quality of the delivered Triple P training moderated outcome with children of Triple P-parents showing less non-aggressive problem-solving than children of parents in the control group when the quality of the training was low. One limitation of this study is that only *n *= 155 out of the *n *= 480 randomized Triple P-families actually attended more than two sessions of the group training leaving the majority of families unexposed to the parenting program (but nevertheless included in the outcome analysis).

Hahlweg, Heinrichs, Kuschel, and Feldmann [22] investigated the six month effectiveness of a therapist-assisted version of the Triple P self-help booklet consisting of 10 chapters [23] for families with preschool-age children in Germany. Sixty-nine families were randomly assigned to either a therapist-assisted self-administered parent training (SDPT+T) or to a waitlist control group (WL). Parents in the SDPT+T received the self-help book and an accompanying video. A Triple P facilitator offered seven telephone consultations which aimed to support parents in skill implementation. Compared to waitlist controls, SDPT+T mothers reported significant short- and six-months reductions in child behavior problems as well as in dysfunctional parenting practices.

Recently, the results of a universal, population based trial to prevent child maltreatment have been published by Prinz, Sanders, Shapiro, Whitaker, and Lutzker [24]. In this study, 18 counties in South Carolina were randomly assigned to either dissemination of the Triple P Positive Parenting Program system or to the services-as-usual control condition, controlling for county population size, poverty rate, and child abuse rate. The referent population were families with at least one child under 8 years. Dissemination involved Triple P training for the existing workforce with over 600 service providers, as well as media and communication strategies. Comparing baseline data in the 5 years before the start of the trial with data after a 2-year period of intervention, significant differences were found for three independently derived population indicators: substantiated child maltreatment (effect size *ES *= 1.09), out-of-home placements (*ES *= 1.22), and child maltreatment injuries (*ES *= 1.14). This study is the first to randomize geographical areas and show preventive impact at a population level.

While these studies used the Triple P interventions with parents of pre- and primary schoolers, Hiscock et al. [4] recruited 733 mothers and investigated whether a three session PT-intervention offered universally in primary care can prevent behavioral problems in 8-month old children over a 24 month time period. At 18 month, there were no significant differences between the intervention and control group. At 24 months, there were no significant differences in externalizing behaviors; however, intervention mothers reported significantly less harsh/abusive parenting and lower unreasonable expectations of child development than control mothers.

Finally, in a controlled study with N = 131 families of preschool children, Lösel, Beelmann, Stemmler, and Jaursch [25] used a German adaptation of the Oregon Social Learning Center parent training [26]. The post and 1-year follow-up teacher ratings showed no significant effects.

In summary, the 20% prevalence rate of child and adolescent DSM-IV-TR disorders is high and internationally comparable. While effective treatments for the disorders have been developed, it seems unlikely that therapy will lower the prevalence rates and certainly not the incidence rates. Low cost preventive interventions seem to be one promising way to achieve the goals of alleviating the burden for children and families. In particular, parent training has been used and evaluated widely; however, randomized controlled trials investigating the efficacy of *universal *prevention are very rare and yielded mixed results. Furthermore, long-term follow-ups of at least two years with parents of pre-school children are nonexistent.

The aims of the current investigation are to evaluate the long-term, two-year efficacy of the group Triple P parenting program administered universally for the prevention of child behavior problems using multi-source assessment, including questionnaires by mother and father, mother-child interaction, and teacher evaluations. Specifically, we hypothesized, that, in contrast to the control group, in the intervention group positive parenting behavior would increase, dysfunctional parenting behaviour, and internalizing and externalizing child behavior would decrease based on parent and teacher ratings.

## Methods

### Recruitment

In the present study, families with children age 3 to 6 years were recruited out of preschools in the city of Braunschweig, Germany. We first contacted all potentially eligible preschools (*N *= 33). Project staff members were present at preschool teacher meetings and explained the project. Twenty-three preschools (70%) expressed interest in participating in the project. Seventeen of these interested preschools were then randomly selected to participate in the project (the others were excluded due to lack of project manpower), and then preschools were randomly assigned to either the intervention or control condition.

### Randomization

**Figure 1:**
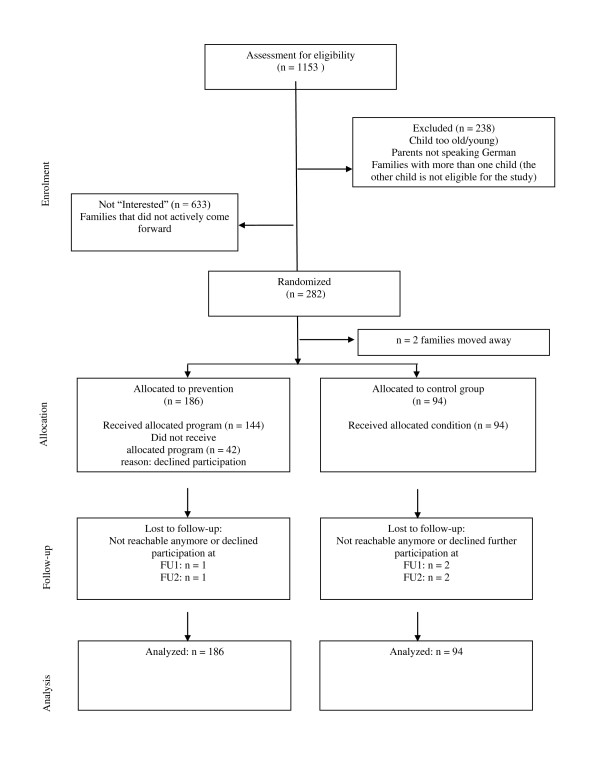
**Flow diagram of the progress through the phases of the randomized Triple P trial**.

We randomized preschools in a 2:1 proportion favouring the intervention group because we anticipated a 50% acceptance rate for the parenting program (for more details see [27]). The project was then presented to the families who received information about the course of the project, the study conditions (developmental/control versus prevention program/experimental), home visit procedures, and financial reimbursement. Interested families could enroll at any time through their preschool. Inclusion criteria were the child's age (2.6 - 6.0 years) and parents' German language ability. The total population consisted of 915 eligible participants; 282 families (31%) enrolled in the project (see Figure 1; modified and extended from [27]). The neighborhood SES was inversely related to participation of families in the project; in low or medium SES areas, only 23%/27% of families participated. In contrast, in neighborhoods with high SES 44% of 280 families participated.

### Participants

Out of the 280 families, 186 were randomized to the intervention and 94 to the control group [28]. The age of the parents ranged between 22 and 47 years (mothers: *M *= 35, *SD *= 5; fathers: *M *= 38, *SD *= 5). The families had between one and four children (*M *= 2.0, *SD *= 0.8). The target children's age averaged 4.5 years (*SD *= 1.0), 51% (*n *= 144) were boys. Seventy-eight percent (*n *= 219) of the couples were married, and 22% (*n *= 61) were single parents (N = 60 single mothers (1 mother participated only in the interview and the behavioural observation but never returned the questionnaires); N = 1 single father). Out of the 219 two-parent families, 3 mothers participated only in the interview and the behavioural observation but never returned the questionnaires. 200 fathers (91% participation rate) completed the questionnaire assessment at pre-test. Fifty-one percent of mothers (62% of fathers) had a High School (= 13 years of school) degree, and 34% (22%) a "Realschule"-degree (= 10 years of school). The family net income was equivalent to the German average, 5% of the families were receiving public assistance, and 7% of mothers (5% of fathers) were immigrants. There were no significant differences between the intervention and the control group in the outcome variables as described below, or in the sociodemographic variables at pretest, with the exception for single parenting: More parents in the control group were single in comparison to the intervention group (CG: *N *= 32, 34.0%, I: *N *= 29, 15.6%; χ^2^(1) = 12.5, p < .001).

Single parenthood is associated with several risk factors (e.g., low income, bad housing), which may impact on the long-term development of children. Therefore, we decided to analyse the data separately for two-parent- and single-parent households. Furthermore, this way of analyzing data allows for the direct comparison of the outcome for mothers and fathers in the same families.

## Measures

### Procedure

The assessments for each family consisted of a battery of self-report questionnaires. At pre-test, two project staff members visited each home for approximately 2-3 hours to complete an interview with a caretaker, conduct a child developmental test [Kaufman Assessment Battery for Children, K-ABC, [29]] and videotape a parent-child interaction task. The self-report measures were completed by both parents in dual-parent families, whereas the interview and the parent-child interaction were usually completed by the mother (97%). At the 1- and 2-year follow up, an interview with the caretaker and the child was conducted. Furthermore, the children completed cognitive tests to assess school performance (not reported here). The multi-method assessment is modelled on other large prevention trial studies, such as Fast Track [30,31]. Compensation for time and effort for the assessments was provided (50 Euro for pre-, and 1-year including the mother-child interaction, 20 Euro for the 2-year follow-up assessments, and 10 Euro for the reduced post-assessment with self-report instruments only); furthermore, parents received feedback about the results of the assessments.

#### Sociodemographic Questionnaire

At pre-assessment, families provided information regarding their age, nationality, exact relationship to the child, education level, employment, receipt of social welfare assistance, and household income. In addition, they provided data on the age and gender of the child of interest and any siblings.

#### Child Behavior Checklist - Parent Report (CBCL 1 1/2 - 5)

The German version of the widely used Child Behavior Checklist (CBCL, [32,33]) consists of 100 items dealing with emotional and behavior problems. The Externalizing Scale of the CBCL assesses conduct problems, such as non-compliance and aggression (Cronbach's *α *in the current sample: mothers: .90/fathers: .92). The Internalizing Scale assesses withdrawal, depression, and anxiety (*α*: .90/.92). At pre-test, the prevalence rate of sub-clinical (T 60 - 63) and clinically relevant (T ≥ 64) children were: Internalizing disorders 18%, Externalizing Disorders 14.5%, and the Total Score 14.5% (maternal rating). Since there are no German norms available for the CBCL 1.5-5, we used the norms provided by Achenbach and Rescorla [32,33]. The present prevalence rates, which are at the lower end of rates found in representative samples [33], indicate that the participants are characteristic for samples in universal prevention studies. At the 2-year follow-up, the CBCL 4-18 was used.

#### Caregiver Teacher Report Form (C-TRF 1.5 - 5)

Pre-school teacher ratings on the Caregiver Teacher Report Form [32] assessed internalizing and externalizing behaviors of children in pre-school. The German version of the C-TRF [34] is analogous to the CBCL in its construction and also contains 100 items. The German version of the C-TRF has been demonstrated to be a reliable and valid instrument. Pre-school teachers received five Euro per child for completing the measure at pre- and the follow-up at 1 year.

#### Parenting Scale (PS, [35])

The German version of the PS was administered to assess parenting skills. The PS is a 35-item questionnaire that measures dysfunctional discipline styles in parents. It yields a total score based on three factors: Laxness (permissive discipline), Over-reactivity (authoritarian discipline, displays of anger, meanness and irritability) and Verbosity (overly long reprimands or reliance on talking). The total score has adequate internal consistency (alpha = .84), good test-retest reliability (*r *= .84), and reliably discriminates between parents of clinic and non-clinic children.

#### Positive Parenting Questionnaire (PPQ)

The 13 item PPQ was adapted from several existing questionnaires e.g., by Strayhorn and Weidman [36] and assesses positive and encouraging parental behaviors (e.g., "I cuddle with my child"). Parents rate the frequencies of their behavior during the most recent two month time period. Answer categories are 0 = never to 3 = very often. Cronbachs *α*'s are .85 for mothers and .87 for fathers.

#### Observation of Mother-Child Interaction

The situations for the parent/child interaction were adapted from McMahon and Estes (Mahon R J, Estes A K: Parent-child interaction task. Observational data collection manuals. Unpublished manuscript, University of Washington, Seattle 1993) and were slightly modified. Mother and child behavior was assessed using a 20-min video recorded home observation at the pre- and 1-year assessment. The observation was divided into four 5-minute tasks recorded consecutively without interruption: (a) child's game/free play, (b) a Lego task, (c) parent and child remained in the same room but completed separate activities, and (d) clean-up. These settings were chosen to replicate a number of experiences that occur regularly in family life. To minimize reactivity effects, observers did not interact with participants and positioned themselves in a minimally obtrusive location.

Observation sessions were coded in 10-second time intervals using the *Revised Family Observation Schedule *(FOS-R-III) [37]. Four composite scores were computed. *Negative child behavior *comprised the percentage of intervals the child displayed negative behavior during the 20-min observation as coded by noncompliance, complaints, aversive demands, physical negative, inappropriate behavior, or interruption. *Positive child behavior *consisted of appropriate verbal interactions, engaged activity of play, and affection. *Negative parent behavior *comprised the percentage of intervals during which the parent displayed negative behavior, namely negative physical contact, aversive question or instruction, aversive attention, or interruption. *Positive parent behavior *was composed of praise, contact, question, instruction, attention, and affection. Five trained observers (mean time needed to be trained: 57 hours) coded the interactions. Each rater coded a selection of interactions from both assessment phases (pre, 1-year-follow-up). All coders were blind to the intervention conditions of the participants, stage of assessment, interactions used for reliability checks, and the specific hypotheses being tested. To maintain reliability, coders rated practice interactions in supervision meetings. Interrater agreement was assessed by having one fifth of the observations randomly selected and coded by a second rater. A satisfactory level of interrater agreement (kappa) was achieved with = .81-.88 for child behavior and = .74-.82 for parent behavior.

### Assessment points

Interview and questionnaire assessments were conducted prior to beginning the parent training (pre-test), after completing the program (post-test), and one and two years after pre-assessment. At the 1- (FU1) and 2-year follow-ups (FU2), three families each dropped out of the study, leaving 274 families (retention rate 99%). Behaviour observations and teacher ratings were conducted only at pre- and FU1 assessment. Unfortunately, we were not able to assess the full TRF sample at FU 1 because *n *= 52 children changed from pre-school to primary school, *n *= 3 children dropped out, and for *n *= 48 children the kindergarten teacher changed, leaving n = 177 TRF-ratings (63%) from *n *= 49 teachers.

### Intervention

The parent training Triple P [18] was introduced to families randomized to the experimental group; the control group was not offered training and was naturally observed for the course of the study. The group parent training format for the experimental condition consisted of four weekly group sessions of two hours each with six to 10 families, and four optional 15-minute phone contacts made on a weekly basis. Parents are taught 17 core child management strategies. Ten of the strategies are designed to promote children's competence and development (e.g., quality time, talking with children, physical affection, praise, setting a good example, behavior charts) and seven strategies are designed to help parents manage misbehavior (e.g., setting rules, directed discussion, planned ignoring, logical consequences, time out). In addition, parents are taught a six-step planned activities routine to enhance the generalization and maintenance of parenting skills (e.g., plan ahead, decide on rules, select engaging activities). Consequently, parents are taught to apply parenting skills to a broad range of target behaviours in both home and community settings with the target child and all relevant siblings. By working through a workbook, parents learn to set and monitor their own goals for behaviour change and enhance their skills in observing their child's and their own behaviour.

In dual-parent families, both parents were invited to participate in program sessions. However, since children did not attend the trainings, dual-parent families usually left one parent with the child(ren) while the other attended the session. Attendance by one parent at one program session was sufficient to be considered as program participation. The attendance rate for program participants was as follows: mothers: 3-4 sessions 88.4%; fathers: 69% none, and only 6,3% attended at least 3 sessions. Attendance rate of telephone contacts: 39% of participants used all four contacts, 13% three; 12% two; 12% one contact; 23% none. It is important to note, that 23% declined the program offer and did not attend at all. As outlined in [27] parents accepting the offer were more likely to report child behaviour problems than did reclining parents.

The satisfaction with the training was assessed from mothers with the Client Satisfaction Questionnaire. Administered at post-intervention only, the 13 items addressed the quality of service provided; how well the program met the parents' needs, increased the parent's skills and decreased the child's problem behaviours; and whether the parent would recommend the program to others. Ninety-one percent were satisfied with the training, 86% liked the atmosphere during the group sessions, and 94% rated the program as helpful.

#### Treatment Integrity

Five female clinical psychologists were trained, licensed, and supervised in the delivery of the interventions. In total, 28 groups were run. In 50% of all group sessions, research assistants completed a protocol adherence checklist, resulting in an adherence to the manual of over 91%. Supervision was provided during regular weekly staff meetings and included the discussion of difficult situations in the group sessions, coaching and conducting role plays with alternative trainer behaviour.

## Results

### Data analysis

The Intention-to-Treat analysis by SPSS 15.0 of the two-years effects consisted of 2 (condition: intervention vs control) by 4 (time: pre, post-intervention, 1 year, 2 year) repeated measures MANOVAs. Significant multivariate effects were followed by univariate ANOVAs. We were most interested in the interaction effect time × group because this effect is most relevant for treatment efficacy. *Intra-group effect-sizes *(ES) were calculated after Rustenbach [38] (M_pre _- M_post, 1, 2 years_)/SD_difference_. The ES was used to show the differential effects in the intervention (ES_I_) and control group (ES_CG_) over time, in particular for the control group to demonstrate the natural course of psychosocial development. *Inter-group ES *(IGES) were calculated by subtracting ES_CG _from E_SI_. The data analysis was conducted a) for two-parent families, separately for mothers and fathers, and b) for single-parent mothers. Missing data were substituted by the "Last Observation Carried Forward"- or the "Last Observation Carried Backwards"-method. The rate of missing data varied dependent on the specific measure and ranged from 2%-9%.

### Long-term efficacy two-parent families

Table 1 shows the means, standard deviations, intra-group, and inter-group effects sizes for the parenting and child measures at pre- and post-intervention, and at the 1 and 2 year follow up for two-parent families.

**Table 1:** Long-term outcome for *two-parent *households.

		Intervention Group				Control Group				IGES		
Variable		Pre	Post	FU1	FU2	Pre	Post	FU1	FU2	Post	FU1	FU2
												
***Mothers***	*Parenting Behavior*											
PPQ	M	2.07	2.16	2.14	2.08	2.08	2.08	2.06	1.99			
	SD	0.37	0.37	0.42	0.41	0.35	0.38	0.38	0.41			
	**ES**	**-**	**-0.14**	**-0.23**	**-0.02**	**-**	**0.02**	**0.10**	**0.32**	**0.16**	**0.33**	**0.34**
PS	M	3.19	2.85	2.85	2.86	3.28	3.27	3.23	3.22			
	SD	0.53	0.64	0.65	0.64	0.54	0.46	0.52	0.47			
	**ES**	**-**	**0.75**	**0.78**	**0.67**	**-**	**0.03**	**0.15**	**0.18**	**0.72**	**0.63**	**0.49**
**Mean IGES (parenting)**										**0.44**	**0.48**	**0.41**
	*Child Behavior (CBCL)*											
Internalizing	M	9.1	7.3	6.7	5.1	6.7	6.2	5.1	4.6			
	SD	6.8	6.0	6.3	5.3	4.4	4.1	3.8	3.4			
	**ES**	**-**	**0.44**	**0.54**	**0.80**	**-**	**0.15**	**0.41**	**0.48**	**0.29**	**0.13**	**0.32**
Externalizing	M	12.2	9.9	8.9	8.4	10.2	9.3	8.4	8.5			
	SD	7.7	6.7	6.8	7.8	6.5	6.6	6.3	6.6			
	**ES**	**-**	**0.50**	**0.62**	**0.62**	**-**	**0.20**	**0.37**	**0.30**	**0.30**	**0.25**	**0.32**
**Mean IGES (child behavior)**										**0.30**	**0.19**	**0.32**
**Mean Total IGES**										**0.37**	**0.34**	**0.37**
*Fathers*	*Parenting Behavior*											
PPQ	M	1.88	1.88	1.88	1.80	1.92	1.95	1.88	1.79			
	SD	0.41	0.43	0.42	0.42	0.37	0.42	0.47	0.45			
	**ES**	**-**	**0.02**	**0.02**	**0.19**	**-**	**0.08**	**-0.10**	**-0.32**	**- 0.06**	**0.12**	**0.51**
PS	M	3.19	3.00	3.00	2.93	3.24	3.23	3.15	3.20			
	SD	0.48	0.54	0.55	0.53	0.40	0.45	0.47	0.49			
	**ES**	**-**	**0.26**	**0.25**	**0.50**	**-**	**0.02**	**0.21**	**0.09**	**0.24**	**0.04**	**0.41**
**Mean IGES (parenting)**										**0.09**	**0.07**	**0.46**
	*Child Behavior (CBCL)*											
Internalizing	M	8.6	6.9	5.9	3.8	7.3	5.8	5.6	3.8			
	SD	6.0	5.8	5.3	3.9	5.3	4.7	4.4	3.2			
	**ES**	**-**	**0.29**	**0.21**	**1.04**	**-**	**0.30**	**0.35**	**-0.32**	**0.00**	**-0.14**	**0.23**
Externalizing	M	11.8	10.2	8.7	7.8	10.9	9.3	9.0	7.6			
	SD	7.2	6.9	6.3	7.1	6.6	7.2	7.1	5.7			
	**ES**	**-**	**0.23**	**0.46**	**0.56**	**-**	**0.23**	**0.28**	**0.54**	**0.00**	**0.18**	**0.02**
**Mean IGES (child behavior)**										**0.00**	**0.02**	**0.13**
												
**Mean Total IGES**										**0.04**	**0.04**	**0.29**

*Note: *Means (M), standard deviations (SD), intra group effect sizes (ES), and inter group effect sizes (IGES) for intervention (I) and control group (CG) for pre, post, 1- and 2-year Follow-up (FU). Sample sizes: mothers: I = 155, CG = 61; fathers: I = 141, CG = 57. PPQ = Positive Parenting Questionnaire, higher scores indicate higher amount of positive parenting; PS = Parenting Scale, higher scores indicate higher dysfunctional parenting.

### Mothers

In the multivariate analysis, a significant time, *F *(12, 203) = 13.0, *p *< .001, a significant group effect, *F *(4, 211) = 6.5, *p *< .001, and a significant group × time interaction, *F *(12, 203) = 3.6, *p *< .001 occurred. In the *univariate *follow-up analyses, *the Positive Parenting Questionnaire PPQ *yielded a significant time effect *F *(3, 642) = 6.3, *p *< .001 and a significant interaction effect group × time *F *(3, 642) = 2.7, *p *= .02. In the *Parenting Scale PS*, a significant time effect *F *(3, 642) = 19.6, *p *< .001, and a significant interaction effect group × time *F *(3, 642) = 12.1, *p *< .001 were found. Similarly, in the *CBCL-Internalizing Scale*, a significant time effect *F *(3, 642) = 31.6, *p *< .001, and a significant interaction effect group × time *F *(3, 642) = 3.3, *p *< .01 were found. For the *CBCL-Externalizing Scale *a significant time effect *F *(3, 642) = 19.9, *p *< .001 and a significant interaction effect group × time *F *(3, 642) = 2.6, *p *= .03 were found. Across all dependent measures, Triple P participants showed significant increases (PPQ) or decreases (PS, CBCL-I, CBCL-E) in comparison to the control mothers.

### Fathers

A multivariate significant time effect, *F *(12, 183) = 20.9, *p *< .0001 and a significant group × time interaction, *F *(12, 183) = 2.2, *p *= .01 resulted. In the *univariate *follow-up analyses, the *Positive Parenting Questionnaire PPQ *yielded a significant time effect *F *(3, 582) = 10.9, *p *< .001. In the *Parenting Scale PS*, a significant time effect *F *(3, 582) = 11.6, *p *< .0001, and a significant interaction effect group × time *F *(3, 582) = 5.5, *p *< .001 were found. In *CBCL-Internalizing Scale*, a significant time effect *F *(3, 582) = 51.6, *p *< .0001 were found. Similarly, for the *CBCL-Externalizing Scale *a significant time effect *F *(3, 582) = 28.8, *p *< .001 were found.

### Effect-sizes

In Table 1 the intra-group (ES) and the inter-group effect sizes (IGES) are depicted for the intervention and control group for each dependent variable. For mothers, the mean IGES for *parenting behavior *at each assessment point showed a slight decline over time (post: 0.43, FU 1: 0.48, and FU 2: 0.41). For fathers, the mean IGES showed an increase over time (0.09, 0.07, and 0.46 respectively). For mothers, the mean IGES for *child behavior *at each assessment point showed a more stable course over time (post: 0.30, FU 1: 0.19, and FU 2: 0.32). For fathers, the mean IGES were very low (0.00, 0.02, and 0.13, respectively).

Finally, mothers demonstrated a mean total IGES of 0.37 (post), 0.34 (FU 1), and 0.37 (FU 2), fathers mean total IGES were 0.04, 0.04, and 0.29 respectively, showing a slight increase over time.

### Long-term efficacy single mothers

Table 2 shows the means, standard deviations, intra-group, and inter-group effects sizes for the parenting and child measures at pre- and post-intervention, and at the 1 and 2 year follow up for *single *mothers. *Multivariate analysis*: A significant time, *F *(12, 46) = 4.7, *p *< .001, a non-significant group effect, *F *(4, 54) = 0.8, *p *= .55, and a non-significant group × time interaction, *F *(12, 46) = 1.5, *p *= .16 resulted.

**Table 2:** Long-term outcome for *single-parent *households:

		Intervention Group				Control Group				IGES		
Variable		Pre	Post	FU1	FU2	Pre	Post	FU1	FU2	Post	FU1	FU2
												
	*Parenting Behavior*											
PPQ	M	2.17	2.17	2.14	2.08	2.16	2.24	2.22	2.06			
	SD	0.42	0.42	0.40	0.43	0.35	0.38	0.38	0.41			
	**ES**	**-**	**-0.02**	**0.07**	**0.22**	**-**	**-0.29**	**-0.18**	**0.30**	**-0.27**	**-0.25**	**-0.08**
PS	M	3.24	3.09	3.01	2.87	3.26	3.08	2.98	2.99			
	SD	0.63	0.67	0.72	0.68	0.65	0.62	0.79	0.81			
	**ES**	-	**0.29**	**0.43**	**0.73**	-	**0.84**	**0.61**	**0.41**	**-0.55**	**-0.18**	**0.32**
**Mean IGES (parenting)**										**-0.28**	**-0.22**	**0.12**
	*Child Behavior (CBCL)*											
Internalizing	M	10.8	10.1	12.1	7.9	10.0	8.1	7.3	4.9			
	SD	8.6	8.5	10.6	7.4	8.5	5.3	5.7	5.1			
	**ES**	**-**	**0.19**	**-0.22**	**0.40**	**-**	**0.49**	**0.55**	**0.98**	**-0.30**	**-0.77**	**-0.58**
Externalizing	M	13.0	13.1	13.4	11.6	12.6	9.5	9.4	8.5			
	SD	8.7	10.1	10.1	9.0	8.5	8.2	8.4	8.1			
	**ES**	**-**	**-0.02**	**-0.06**	**0.17**	**-**	**0.73**	**0.58**	**0.73**	**-0.75**	**-0.64**	**-0.56**
**Mean IGES (child behavior)**										**-0.53**	**-0.71**	**-0.57**
												
**Mean Total IGES**										**-0.41**	**-0.43**	**-0.35**

*Note: *Means (M), standard deviations (SD), intra-group effect sizes (ES), and inter-group effect sizes (IGES) for intervention (I) and control group (CG) for pre, post, 1- and 2-year Follow-up (FU). Sample sizes: I = 28, CG = 31. PPQ = Positive Parenting Questionnaire, higher scores indicate higher amount of positive parenting; PS = Parenting Scale, higher scores indicate higher dysfunctional parenting.

#### Effect Sizes

In Table 2 the intra-group (ES) and the inter-group effect sizes (IGES) are depicted for the intervention and control group. The mean IGES for *parenting behavior *showed - unexpectedly - negative IGES for post and FU 1: -0.41, -0.22, and at FU 2: 0.12. The mean IGES for *child CBCL-behavior *showed the same pattern: -0.53, -0.71, and -0.57 at FU 2. The mean total IGES were -0.47 (post), -0.47 (FU 1), and -0.35 (FU 2).

### Behavioral Observation and teacher ratings for two-parent families

Table 3 shows the means, standard deviations, intra-group, and inter-group effects sizes for the *Behavioral Observation FOS *variables and the Teacher TRF ratings at pre- and 1-year follow up for two-parent families. In the multivariate analysis of the *behavioral observation data (FOS)*, a significant time, *F *(4, 205) = 8.7, *p *< .001, a significant group effect *F *(4, 209) = 2.4, *p *= .03, and a non-significant group × time interaction, *F *(4, 205) = 0.2, *p *< .47 occurred. Intra-group effect sizes ranged from -0.04 to 0.00. In the *TRF teacher ratings*, in the multivariate analysis, a significant time, *F *(2, 143) = 7.2, *p *< .001, a non-significant group effect *F *(2, 143) = 1.8, *p *= .085, and a non-significant group × time interaction, *F *(2, 143) = 1.3, *p *< .14 occurred.

**Table 3:** One year outcome for two- and one parent households

		Intervention Group		Control Group		IGES
Variable		Pre	FU1	Pre	FU1	FU1
**Two Parent Households**						
*Behavioral Observation (FOS)*						
Positive Mother	M	0.18	0.17	0.17	0.17	
	SD	0.03	0.04	0.03	0.03	
	**ES**	**-**	**0.35**	**-**	**0.39**	**-0.04**
Negative Mother	M	0.003	0.002	0.003	0.003	
	SD	0.005	0.003	0.004	0.004	
	**ES**	**-**	**0.04**	**-**	**0.06**	**-0.02**
Positive Child	M	0.30	0.32	0.30	0.31	
	SD	0.05	0.04	0.05	0.04	
	**ES**	**-**	**-0.34**	**-**	**-0.34**	**0.00**
Negative Child	M	0.03	0.02	0.04	0.02	
	SD	0.04	0.03	0.04	0.03	
	**ES**	**-**	**0.39**	**-**	**0.42**	**-0.03**
**Mean IGES**						**-0.03**
*Caregiver Teacher Ratings C-TRF*						
Internalizing	M	6.78	5.06	6.86	4.96	
	SD	5.61	5.17	6.04	3.97	
	**ES**	**-**	**0.29**	**-**	**0.32**	**-0.03**
Externalizing	M	7.78	6.65	11.30	7.80	
	SD	9.01	9.14	10.09	6.21	
	**ES**	**-**	**0.12**	**-**	**0.36**	**-0.24**
**Mean IGES**						**-0.14**
						
**Single Parent Households**						
*Behavioral Observation (FOS)*						
Positive Mother	M	0.18	0.16	0.17	0.17	
	SD	0.03	0.02	0.03	0.03	
	**ES**	**-**	**0.49**	**-**	**0.17**	**0.32**
Negative Mother	M	0.003	0.003	0.003	0.003	
	SD	0.003	0.005	0.004	0.004	
	**ES**	**-**	**0.03**	**-**	**-0.05**	**-0.02**
Positive Child	M	0.29	0.32	0.31	0.32	
	SD	0.06	0.03	0.04	0.04	
	**ES**	**-**	**-0.46**	**-**	**-0.23**	**0.23**
Negative Child	M	0.04	0.01	0.02	0.02	
	SD	0.04	0.02	0.02	0.03	
	**ES**	**-**	**0.65**	**-**	**0.11**	**0.54**
**Mean IGES**						**0.27**
*Caregiver Teacher Ratings C-TRF*						
Internalizing	M	11.07	9.50	6.77	8.00	
	SD	9.26	7.12	7.12	7.40	
	**ES**	**-**	**0.26**	**-**	**-0.21**	**0.47**
Externalizing	M	7.78	6.65	11.30	7.80	
	SD	9.01	9.14	10.09	6.21	
	**ES**	**-**	**-0.09**	**-**	**-0.15**	**-0.06**
**Mean IGES**						**0.22**

*Note: *Based on behavioral observation (Family Observation Schedule FOS; Sanders et al., 2000) and caregiver teacher ratings (TRF; Achenbach & Rescorla, 2000): Means (M), standard deviations (SD), intra group effect sizes (ES), and inter group effect sizes (IGES) for intervention (I) and control group (CG) for pre and 1- year Follow-up (FU). Sample sizes: two parent households: Behavioral Observation FOS: I = 154, CG = 60; Teacher Ratings TRF: I = 102, CG = 44. Sample sizes: single parent households: Behavioral Observation FOS: I = 28, CG = 31; Caregiver Teacher Ratings C-TRF: I = 14, CG = 17.

### Behavioral Observation and teacher ratings for single mother families

Table 3 shows the means, standard deviations, intra-group, and inter-group effects sizes for the Behavioral Observation FOS variables and the Teacher C-TRF ratings at pre- and 1-year follow up for single-parent families. In the multivariate analysis of behavioural observation data, a significant time, *F *(4, 54) = 5.0, *p *< .001, a non-significant group effect *F *(4, 54) = 0.7, *p *= .31, and a significant group × time interaction, *F *(4, 54) = 2.5, *p *< .03 occurred. In the *Teacher Rating C-TRF*, the multivariate analysis showed non-significant results.

#### Univariate effects

In the FOS - *Positive Mother Behaviour*, a significant time effect *F *(1, 57) = 10.4, *p *= .001, and a significant interaction effect group × time *F *(1, 57) = 2.7, *p *= .054 were found (IGES = 0.32). For the FOS-*Negative Mother Behavior *non-significant effects resulted (IGES = -0.02). For the FOS *Positive Child Behavior *a significant time effect *F *(1, 57) = 8.3, *p *= .003 were found (IGES = 0.23). For the FOS *Negative Child Behavior *a significant time effect *F *(1, 57) = 11.5, *p *= .000 and a significant interaction effect group × time *F *(1, 57) = 5.5, *p *= .011 were found (IGES = 0.54).

## Discussion

The aims of the study were to evaluate the long-term, two-year efficacy of the group Triple P parenting program administered universally using multi-source assessment, including questionnaires by mother and father, mother-child interaction, and teacher evaluations. Furthermore, the efficacy of the intervention for mothers, fathers, and single parents were evaluated. We hypothesized, that, in contrast to the control group, in the intervention group positive parenting behavior would increase, dysfunctional parenting behavior, internalizing and externalizing child behavior would decrease. These hypotheses were partially supported.

At the 2-year follow-up, both parents in the Triple P intervention reported significant reductions in dysfunctional parenting behavior, and mothers also an increase in positive parenting behavior. In addition, mothers reported significant reductions in internalizing and externalizing child behavior. Single-parent mothers in the Triple P intervention did not report significant changes in parenting or child problem behavior which is primarily due to inexplicable high positive effects in single parent mothers of the control group. Neither mother-child interactions nor teacher ratings yielded significant results.

The current findings, that over 90% of the participants were satisfied with the training and rated it as helpful, add to the notion that Triple P is well regarded by parents and can be applied also in non-English speaking countries like Germany or Switzerland [39].

### Two parent families

For mothers, the mean Inter-Group Effect Size IGES for *parenting behavior *remained stable over time: from 0.43 at post to 0.41 at FU 2-years, while for fathers, the mean IGES showed an increase and were 0.09 and 0.46 respectively. Thus, the hypotheses for an increase of positive parenting behavior and a decrease in dysfunctional parenting behavior were completely supported for mothers and partially supported for fathers. It is interesting to see the increase in parenting competency for fathers despite the fact that only about 7% of fathers participated regularly in the groups. This may be due to regular parental communication about program components when mothers returned from the session and informed fathers about the content. For mothers, the mean IGES for *child CBCL-behavior *showed also a stable course over time: 0.30 at post and 0.32 at the 2-year FU. For fathers, the mean IGES were very low (0.0 and 0.13 respectively). Thus, the hypothesis of a decrease in child behavior problems was only supported for mothers.

The finding, that the effects for fathers at post and follow-up 1-year later are much weaker than mothers is congruent with previous findings e.g., by Sanders et al. [40] or by Bodenmann et al. [39]. Unexpectedly, at the 2-year follow-up, fathers caught up with mothers, who achieved a mean total IGES of 0.37 at post and of 0.37 at FU-2, while fathers mean IGES were 0.04 and 0.29 respectively. These increases over time could tentatively be explained by modeling effects in the family. However, more research is clearly necessary to explain these results in more detail and to find ways to engage more fathers to participate in the training. In general, our effect sizes for mothers compare very well with the meta-analytic findings of Kaminski et al. [14] and Nowak and Heinrichs [18], who reported a mean effect size for parenting interventions of 0.34 - 0.42 for pre-post comparisons. Our findings indicate that the results are even stable over a two-year time span.

### Single mothers

There were no significant multivariate changes for the parenting and child measures at pre- and post-intervention, and at the 1 and 2 year follow up. This may be due to the small sample size and the resulting statistical power restrictions. However, looking at the effect-sizes, unexpectedly, negative effect sizes resulted. These negative effect sizes resulted from control group mothers showing an increase in parenting competencies and a decrease in child behaviour problems over the two years of the project. It is unclear how this pattern emerged. Potentially, single parent households who did not participate in a parent training did self-evaluate repeatedly and might have concluded that they do not do so badly. Single parents in the intervention group may, in contrast, felt challenged in the parenting groups with other (non-single) mothers when comparing their situation with intact families. The result hints at the importance to separate single- and two-parent households in their response to an intervention and to also attend to a potential interaction effect between parent status and parenting intervention format.

Some support for a relative comparison theory comes from the behavioral observation data. Contrary to our assumptions, there were no significant changes in the 2-parent families for the mother or child interaction behaviours with an IGES of -0.03 at the 1-year follow-up. However, for single parent families, Triple P mothers showed an increase in positive behaviour and a decrease in negative child behaviour, resulting in a mean total ES of 0.27.

Thus, while in self-report measures we did not find significant changes, in clinician-rated data there is a significant change. To the contrary, in two-parent households we found significant changes in self-report measures but little evidence was established for changes in clinician-rated data. There are a few explanations that may account for this pattern of results. This is a universal prevention study and only about 15% of children exhibited externalizing behaviour in the clinical range. Therefore the observed levels of negative behavior were low; a floor effect may have been operating precluding the demonstration of intervention effects. In fact, single-parent households had higher observed levels of negative behavior at pre-assessment than two-parent households. Furthermore, there were low correlations between observed and parent-reported child behavior, indicating that perhaps the observational tasks were not capturing an adequate sample of children's behavior. There seems to be a need to develop program variants that work more effectively with single parents.

### Limitation of observational methods

It is important to note the limitation of observational methods when dealing with behaviors that are high amplitude but relatively low rate. However, it is a drawback of the study that the results were primarily demonstrated through maternal self-report given that there can be biases in parental reports. In retrospect, the selection of a different set of observational tasks (e.g., family mealtime) may have provided a more change-sensitive index of parent-child interaction [41]. However, not every research question requires observational data and this method is neither a panacea nor does it necessarily provide more valid findings than other forms of measurement including parent reports. For example, observational methods can be impractical in large scale population trials, they are intrusive (video observation is the most frequently stated reasons for families to not participate in a research project, specifically in socially disadvantaged neighborhoods [42]) thus contributing to subject attrition and biased non-representative samples of families.

### Teacher ratings

Finally, there were also no significant results for the two parent or single parent families in teacher ratings. This may be due to the low power because of the 38% drop-out rate for various reasons, mainly that kindergarten children changed to primary school or that the kindergarten teacher changed. This drop-out rate corresponds with another German study by Lösel et al. [25], who reported a 50% rate and also no significant effects at post and the 1-year follow-up. Non-significant effects for teacher ratings are common in the literature, even in studies of selected [e.g., [43]] or indicated prevention [31,44] no significant time × group interaction with higher base rates of externalizing behaviour occurred indicating that a floor effect may not play a crucial role in this lack of findings.

### Strengths of study

The study had several strengths. First, it is the only universal efficacy study with preschool children conducted with a 2-year follow-up and with an untreated control group over that time span. Second, the retention rate at the 2-year FU of 99% is very high. Third, for the analysis the original randomization was retained, in that the Triple P decliners were kept in the Triple P group, a bias against the hypotheses. Fourth, an Intention-to-Treat analysis was used. Fifth, mothers and fathers, and single parents were included and analyzed separately, allowing differential interpretation of the results. However, the study is also limited.

### Limitations of study

The conclusions rely solely on self report, because behavioral observation and teacher ratings resulted in non-significant findings for two-parent households. The sample size was too small to look at incidence rates which would have been the best way to establish a true prevention effect. And the sample is relatively advantaged with only 1/3 of all potentially eligible families participating. For some of the sub-sample analysis (single mothers, teacher ratings) the study was restricted in statistical power, e.g. for the single mother comparisons for medium effect sizes to be detected as statistical significant the power was 36%. In the absence of sufficient power it remains unclear whether the conclusions that parenting programs are of no benefit to sole parents in groups with mothers from two-parent households or fail to produce outcomes in school settings is warranted. Further research is urgently called for to answer these important questions. Furthermore, we did not stratify and used separated randomisation for single- and two-parent households which future studies should consider.

## Conclusions

The primary goal for universal prevention should be focused on increasing recruitment and engagement of families in prevention programs and not so much in developing new programs which may or may not be effective. In terms of efficacy, there is sufficient data to assume that parenting programs are efficacious in changing self-reported parenting and maternal-reported child behavior problems. Many prevalence studies rely on this type of data and thus, it seems appropriate to conclude that universal prevention is worth the effort.

## Competing interests

The authors declare that they have no competing interests.

## Authors' contributions

KH and NH have made substantial contributions to the conception, design, statistical analysis, interpretation of the data, and writing of the paper. AK, HB, SN have made substantial contributions to the acquisition of data and supervision of data collection as well as to analyzing the behavior observation data. All authors read and approved the final manuscript.
